# Dual effects of slightly acidic electrolyzed water on rice seed germination: insights from water dynamics via low-field NMR

**DOI:** 10.3389/fpls.2026.1740753

**Published:** 2026-03-06

**Authors:** Tiangang Hou, Fangming Cui, Mengnan Yang, Wenzhong Zhang, Jiping Gao, Mingjin Xin, Hongguang Cui, Cuihong Liu, Liyan Wu

**Affiliations:** 1 College of Engineering, Shenyang Agricultural University, Shenyang, China; 2 College of Information and Electrical Engineering, Shenyang Agricultural University, Shenyang, China; 3 College of Agriculture, Shenyang Agricultural University, Shenyang, China

**Keywords:** electrolyzed water, nuclear magnetic resonance, rice, signal amplitude, transverse relaxation time, water

## Abstract

To investigate the effects of slightly acidic electrolyzed water (SAEW) treatment on rice seed germination, rice seeds were soaked with SAEW at different available chlorine concentrations (ACC) of 10, 20, 30, 40, 50, and 60 mg/L. A standard seed germination test was conducted to summarize the influence of SAEW with varying ACC on rice seed germination. Meanwhile, low-field nuclear magnetic resonance (LF-NMR) and magnetic resonance imaging (MRI) technologies were employed to reveal the underlying mechanisms by studying the internal moisture changes, water migration patterns, and water distribution characteristics. The results demonstrated a hormetic effect of SAEW on rice seed germination, characterized by significant promotion at lower ACCs (10–40 mg/L) and inhibition at higher ACCs (50–60 mg/L). The most pronounced promotive effect was observed at an ACC of 30 mg/L. Low ACC SAEW accelerated the water storage rate within the rice seeds, facilitated the conversion and accumulation of free water, thereby providing favorable moisture conditions for seed germination and subsequently promoting rice growth. In contrast, high ACC SAEW damaged the rice cell walls under osmotic stress, leading to a reduced water absorption rate and consequently inhibiting rice growth. This study, starting from the pre-treatment of rice seeds, investigated the entire process, providing theoretical support and data reference for rice production and processing.

## 1 Introduction

Rice, as a common cereal crop, is extensively cultivated across Asian countries and serves as a staple food highly favored by Asians (Atique Ur et al., 2012). Increasing rice yield and production has consistently attracted significant attention. As one of China’s primary grain crops, the high quality and yield of rice are closely linked to seed vigor (Son, 2013). Seed vigor directly determines the quality traits of seedling emergence and establishment—higher vigor correlates with stronger stress resistance, faster and more uniform emergence, and higher germination rates, reflecting greater potential for high yield and quality (Huang et al., 2024). Thus, seed vigor fundamentally influences crop growth and final productivity. Within modern mechanized rice production systems, the fundamental importance of premium seeds, especially those possessing high vigor, has been increasingly recognized for their indispensable role in cultivating ideal plant populations and maximizing grain yield (Xie et al., 2022).

Electrolyzed water, recognized for its high biosafety, efficient sterilization, absence of residue, ease of production, and low operational cost, has been widely applied in grain production (Li et al., 2019). It is generated by electrolyzing a solution of NaCl or HCl at a specific concentration in an electrolytic cell, which alters key parameters such as oxidation-reduction potential (ORP), pH, and available chlorine concentrations (ACC), resulting in the production of both acidic electrolyzed water and alkaline electrolyzed water, each possessing distinct physiological functions (Tyagi et al., 2022; Li et al., 2023). Its primary advantage lies in ensuring microbial safety during grain germination without adversely affecting sprout growth, while also enriching bioactive components in germinated grains (Liang et al., 2019; Rao et al., 2022). Previous studies have shown that soaking tartary buckwheat seeds in slightly acidic electrolyzed water (SAEW) a pH of 5.9 and an ACC of 19.5 mg/L led to the enrichment of total phenols, total flavonoids, and rutin throughout the germination period. The maximum accumulation of these compounds occurred on day 9, reaching 32.27 mg/g, 23.97 mg/g, and 19.96 mg/g, respectively. Additionally, SAEW with a pH of 5.83 and an ACC of 20.3 mg/L promoted the accumulation of γ-aminobutyric acid (GABA) in germinated buckwheat. Over an 8-day observation period, the GABA content in buckwheat sprouts increased from 0.10 mg/g to 1.43 mg/g (Li et al., 2015). Thus, SAEW presents a promising method for seed production. However, research on the mechanism by which SAEW promotes the growth of rice seeds remains limited both domestically and internationally.

Low-field nuclear magnetic resonance (LF-NMR), as an emerging detection technique in the submicroscopic domain, is characterized by its short sampling time, operational simplicity, non-destructive nature, environmental friendliness, and high measurement accuracy (de Moraes et al., 2025). It is widely applied in fields such as agriculture, food science, biology, medicine, physics, and petrochemical engineering, serving as an ideal method for quantifying water content and monitoring migration patterns in crops (Guo et al., 2024). LF-NMR leverages the spin relaxation properties of hydrogen nuclei under a magnetic field to investigate changes in relaxation times at the molecular level, thereby inferring alterations in the internal water state and translocation dynamics of tested materials (Lahaye et al., 2015). Although LF-NMR has been extensively utilized in seed water research, few studies domestically or internationally have employed this technology to explore the impact of electrolyzed water on rice seed vigor. Owing to its non-invasive and non-destructive attributes, LF-NMR can effectively monitor water absorption and distribution dynamics during rice seed germination, providing a robust tool for elucidating the mechanistic links between water status and seed physiological activity (Kamal et al., 2019).

**Figure 1:**
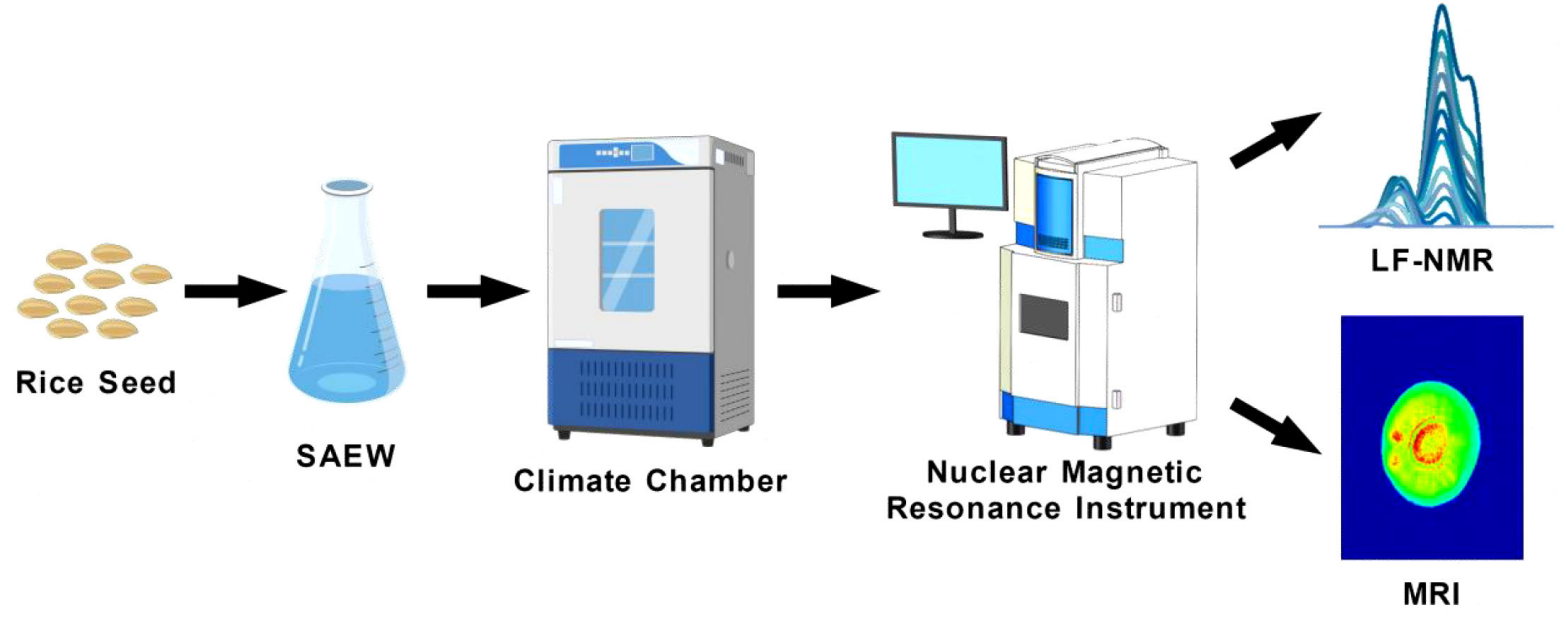
Experimental flowchart for LF-NMR analysis of rice seeds treated with SAEW.

This study employed LF-NMR technology to systematically evaluate the impact of SAEW with varying ACC on the germination dynamics of *Oryza sativa* L. cv. Nipponbare. Rice seeds were subjected to immersion and periodic spraying with SAEW solutions at different ACC levels, followed by cultivation under controlled artificial conditions simulating optimal growth environments. Throughout the experiment, LF-NMR spectroscopy and imaging data were collected to monitor internal water dynamics, while parallel assessments of physicochemical indicators were conducted. The integrated approach aimed to elucidate the mechanisms underlying SAEW-induced effects on seed germination, providing a theoretical foundation and empirical data to support the rational application of SAEW in rice production practices. The experimental workflow is summarized in **Figure 1**.

## 2 Materials and methods

### 2.1 Experimental instruments and materials

#### 2.1.1 Experimental instruments

The following instruments were used in this study:

1. Low-Field Nuclear Magnetic Resonance Analyzer (Model: NMI20-015V-I, Shanghai Nuimei Electronic Technology Co., Ltd., China). The analyzer features a permanent magnet generating a magnetic field strength of 0.50 T and operates at a radiofrequency pulse of 21 MHz. The probe is equipped with a 15-mm diameter coil, and the magnet temperature was maintained at 32 °C during measurements.
2. Analytical Balance (Model: BSA124S-CW, Sartorius, Germany). The balance has a maximum capacity of 120 g with a precision of 0.1 mg.
3. Intelligent Artificial Climate Incubator (Model: RTOP-268D, Zhejiang Top Instrument Co., Ltd., China). The incubator provides a temperature range of 0–50 °C (accuracy: ± 0.5 °C) and a relative humidity range of 50–95% (fluctuation: ± 5%).
4. pH/ORP Meter (Model: YHBJ-262, Shanghai Leici Instrument Science and Technology Co., Ltd., China). The meter measures pH in the range of -2.00 to 20.00 (accuracy: ± 0.01) and oxidation-reduction potential (ORP) from -2000.0 to 2000.0 mV (accuracy: ± 0.3 mV).

#### 2.1.2 Experimental materials

The experimental material was Japanese rice seeds (*Oryza sativa* L. cv. Nipponbare). The seeds were harvested on September 10, 2024, in Wuhan City, Hubei Province, China. Immediately after harvest, the seeds were sealed in airtight polyethylene bags and stored at 4 °C to maintain seed viability and physiological integrity until the experiments began.

### 2.2 Preparation of SAEW

SAEW was generated using a laboratory-scale, membrane-electrode assembly (MEA) electrolyzer, which was developed in-house. The electrolysis process was performed by electrolyzing a dilute sodium chloride solution (0.1% w/v) using direct current (DC) power supply set at a constant current of 1.5 A for a duration of 5 minutes. The generated SAEW was immediately collected in polypropylene containers to minimize the degradation of active components and used promptly (Liu et al., 2014).

To obtain SAEW solutions with varying physicochemical properties, the initially produced concentrated SAEW solution (electrolyte) was diluted with deionized water to achieve target ACC of 10, 20, 30, 40, 50, and 60 mg/L. The pH and ORP of the SAEW solutions were measured using a calibrated pH/ORP meter. The ACC was quantitatively determined by the standard iodometric titration method. The key physicochemical parameters of the prepared SAEW solutions are summarized in **Table 1**.

**Table 1:** Physicochemical parameters of SAEW.

Treatment	pH	ORP (mV)	ACC (mg/L)
CK	7.15 ± 0.03 b	394.3 ± 3.05 a	ND
SAEW10	5.80 ± 0.05 a	911.4 ± 3.33 b	10.11 ± 0.13 a
SAEW20	5.74 ± 0.04 a	927.7 ± 1.98 b	19.98 ± 0.21 b
SAEW30	5.77 ± 0.02 a	925.5 ± 2.18 b	30.04 ± 0.17 c
SAEW40	5.82 ± 0.05 a	916.9 ± 3.89 b	40.11 ± 0.19 d
SAEW50	5.79 ± 0.04 a	930.1 ± 2.55 b	49.95 ± 0.15 e
SAEW60	5.84 ± 0.03 a	923.8 ± 3.88 b	60.06 ± 0.16 f

Values are expressed as mean ± SD. ORP: oxidation-reduction potential (mV); ACC: available chlorine concentration (mg/L); CK: deionized water; SAEW: slightly acidic electrolyzed water. Numbers 10, 20, 30, 40, 50, and 60 represent SAEW with ACC values of approximately 10, 20, 30, 40, 50, and 60 mg/L, respectively. ND: not detected. Data are expressed as means ± SE. Means with the same letter are not significantly different at *P* < 0.05 according to Duncan’s Multiple Range Test.

### 2.3 Sample preparation and treatment

Plump and uniform rice seeds were selected for LF-NMR spectroscopy, magnetic resonance imaging (MRI), and germination tests. The seeds were first rinsed three times with corresponding, SAEW, or deionized water (control), followed by immersion in the respective solutions. A 12 h + 12 h + 12 h soaking protocol was adopted: seeds were immersed for 12 h, air-dried for 12 h, and then re-immersed for another 12 h (Hou et al., 2025). After treatment, the seeds were evenly placed on a germination bed consisting of two layers of filter paper in 115 × 115 mm plastic germination boxes, with an appropriate amount of the corresponding treatment solution added. All samples were incubated in an artificial climate chamber set at a temperature of 28 ± 1 °C, relative humidity of 85 ± 2% RH, and a light/dark cycle of 12 h:12 h. During the incubation period, 50 ml of the corresponding treatment solution was sprayed onto the seeds every 12 hours. Germination indicators and LF-NMR data were recorded every 24 hours over a 7-day period.

### 2.4 Low-field magnetic resonance imaging test

Proton density-weighted images of rice seeds in the coronal plane were acquired using the Spin Echo (SE) pulse sequence within the MRI imaging software. The key imaging parameters were set as follows: repetition time (TR) = 300 ms, echo time (TE) = 5.885 ms, and number of signal averages (Averages) = 32.

**Figure 2:**
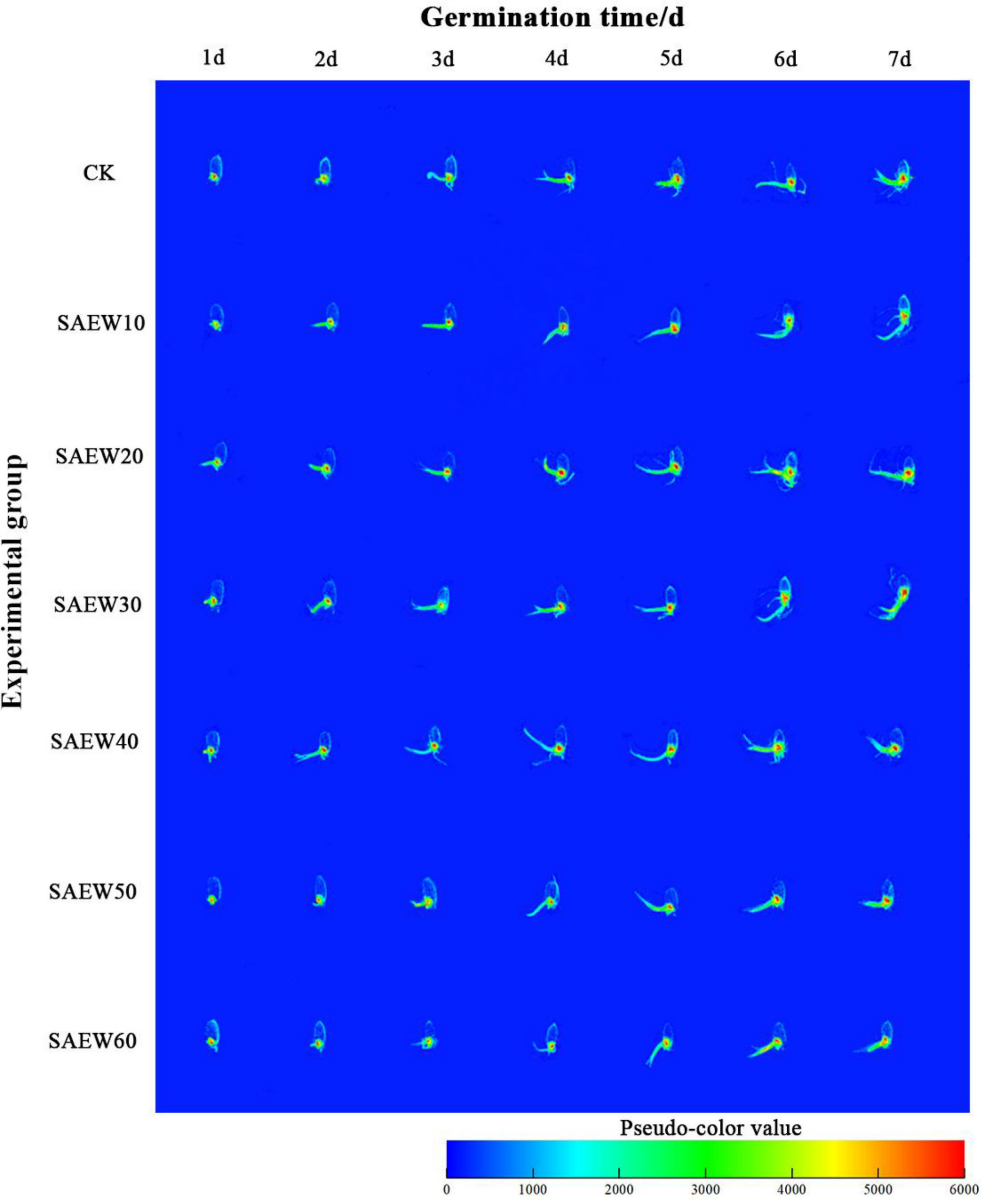
Pseudo-color diagram of the germination process of rice seeds soaked in SAEW from different ACCs.

The low-field MRI experiment assessed internal water distribution in rice seeds by detecting changes in hydrogen proton content. Proton density-weighted images acquired during the experiment underwent uniform grayscale mapping and filtering, followed by pseudo-color processing to enhance visual clarity (Penfield, 2017; Li et al., 2020). In **Figure 2**, red areas indicate regions with high hydrogen proton density, corresponding to elevated water retention capacity in rice seeds; blue areas reflect low proton density and reduced water retention, based on this to determine the pattern of moisture distribution (Kim et al., 2018; Plutenko et al., 2024).

### 2.5 Low-field nuclear magnetic resonance spectroscopy test

Prior to the LF-NMR spectroscopy experiments, each group of seeds was weighed separately to facilitate subsequent data normalization. A free induction decay (FID) pulse experiment was first conducted on a standard oil sample to ensure accurate resonance excitation of spin nuclei and reliability of data outcomes in subsequent experiments. The transverse relaxation time (*T*_2_) of rice seeds was detected using the Carr-Purcell-Meiboom-Gill (CPMG) pulse sequence. After inversion, the *T*_2_ relaxation spectrum of the seeds was obtained, including relaxation times and corresponding signal amplitudes. The CPMG sequence parameters were configured as follows: a 90° pulse width (P1) of 7 μs, an echo time (TE) of 14 μs, 64 scans (NS), a repetition time (TW) of 2,000 ms, and 3,000 echoes (NECH) acquired starting at 0.01 ms. The inversion process utilized 100,000 iterations with a cut-off time of 10,000 ms.

### 2.6 Determine the germination indicators of rice seeds

In the standard germination test, the number of germinated rice seeds (defined by radicle protrusion ≥ 2 mm) in each experimental group is recorded every day. The germination rate is represented by the ratio of the number of germinated seeds in the experimental group to the total number of seeds in the group. The fresh weight of each group of samples was also measured periodically. On the 7th day of the experiment, the root length and shoot length of the germinated rice seed samples were measured using a digital vernier caliper.

### 2.7 Statistical analysis

To eliminate the influence of initial mass variations among experimental samples on the results, the *T*_2_ inversion data of rice seeds collected via LF-NMR were normalized. The normalized inversion parameters of the seeds were subsequently subjected to variance analysis using SPSS 25 software (SPSS Inc, USA), and differences among treatments were further compared by Duncan’s multiple comparison test at a significance level of *P* < 0.05. Data visualization was performed with Origin 2021 software (Microcal, USA). All measurements were repeated three times, and the average values were used for analysis. Proton density-weighted images of rice seeds were processed with pseudo-color rendering using Niumai NMR image processing software to enhance visual distinction of internal water distribution characteristics.

## 3 Results

### 3.1 Analysis of low-field MRI test results

With prolonged seed germination time, **Figure 2** presents pseudocolor images depicting the growth process of rice seeds soaked in SAEW with different ACC levels. The pseudocolor images reveal that throughout the germination process, the high-brightness areas in seeds treated with SAEW10, SAEW20, SAEW30, and SAEW40 consistently exceeded those in the CK group as well as the SAEW50 and SAEW60 treatment groups. Notably, compared with the other treatment groups, seeds in the SAEW30 treatment group exhibited the most extensive high-brightness regions, indicating the highest water-holding capacity of rice seeds under this condition.

### 3.2 Analysis of the results of low-field nuclear magnetic resonance spectroscopy tests

#### 3.2.1 Low field NMR *T*_2_ relaxation spectrum

**Figure 3:**
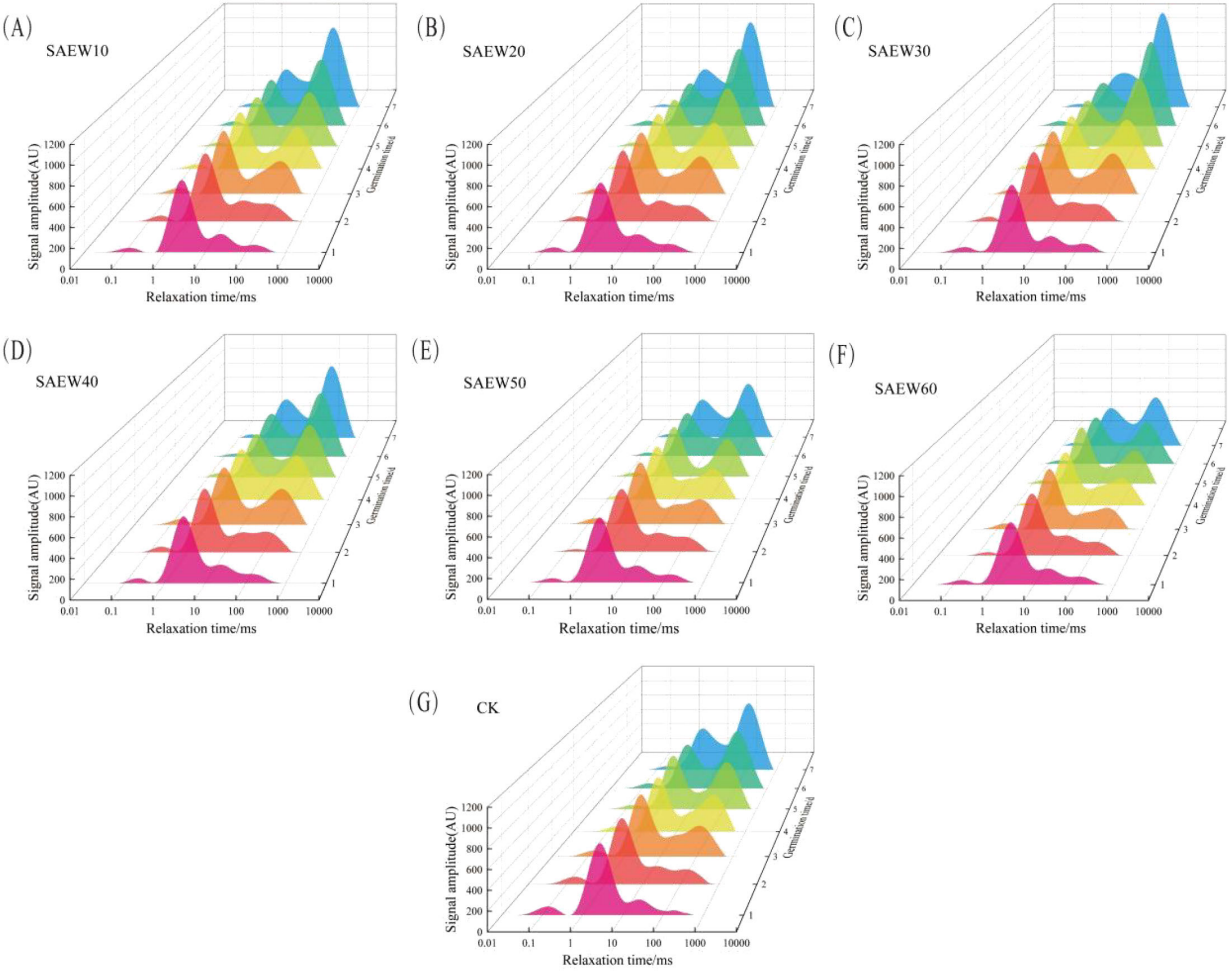
*T*_2_ relaxation spectra of rice seeds in seven experimental groups. **(A)** SAEW10; **(B)** SAEW20; **(C)** SAEW30; **(D)** SAEW40; **(E)** SAEW50; **(F)** SAEW60; **(G)** CK.

The transverse relaxation time (*T*_2_) reflects the mobility and distribution of hydrogen protons within a sample, providing an accurate indication of changes in water content, physical state, and migration patterns inside the sample (Yang et al., 2019). By observing variations in the bound state of water, the influence of SAEW on water enrichment within germinating rice seeds can be investigated. The individual peaks in the *T*_2_ relaxation spectrum correspond to different states of water in the sample. The length of the relaxation time indicates water mobility: longer relaxation times signify greater mobility (Xing et al., 2021). The *T*_2_ relaxation spectra for the seven experimental groups of rice seeds are shown in **Figure 3**. Based on the differences in transverse relaxation time *T*_2_, the water inside rice seeds is categorized into three distinct states: The shortest relaxation time, *T*_21_ (0.01–1 ms), is attributed to bound water, which is tightly associated with intracellular organic matter. This water exists inside rice cells, bound to proteins and other components via hydrogen bonds. These bonds are strong and stable, preventing the water from moving freely and preventing it from participating in metabolic processes. This component also includes signals from rapidly decaying macromolecules such as proteins and amino acids (Wang et al., 2025). The shorter relaxation time, *T*_22_ (1–10 ms), represents semi-bound water retained within structures. This water is weakly hydrogen-bonded to amide groups and hydroxyl groups in the rice, making the bonding less stable. The signal for this component overlaps with the signal from oil. Since the variation in oil content in this experiment had a minor impact, all changes in peak area were considered to be caused by changes in semi-bound water content (Lin et al., 2018). The longest relaxation time, *T*_23_ (10–1000 ms), corresponds to free water that moves within intracellular and intercellular spaces. This water exists in the internal gaps of rice seeds via capillary action, exhibits high mobility, acts as a good solvent, and its abundance indicates more vigorous metabolic activity (Li et al., 2020). The peak area in the *T_2_* relaxation spectrum reflects water content. A larger peak area indicates a higher content of water in that particular state. The peak areas corresponding to the three water states are denoted as *A*_21_, *A*_22_, and *A*_23_, respectively. The total water content, represented by *A*, is the sum of these three values (*A* = *A*_21_ + *A*_22_ + *A*_23_).

As illustrated in **Figure 3**, Duncan’s multiple range test analysis of the *T*_2_ values indicated that the *T*_21_ values did not exhibit significant alterations across all experimental groups (*P* > 0.05). In contrast, a significant rightward shift in *T*_22_ values was observed during the later stages of rice seed germination (*P* < 0.05). Furthermore, the *T*_23_ values in all experimental groups also shifted to the right to varying degrees. Compared to the CK group, the SAEW30 treatment group exhibited the most pronounced rightward shift, suggesting that treatment with SAEW of a lower ACC can increase the degree of freedom of water molecules inside the rice seeds and enhance metabolic activity, thereby promoting seed germination. The *T*_23_ values of the SAEW50 and SAEW60 groups were lower than those of the CK group, indicating a more restricted state of water within the seeds. This restriction of water mobility is indicative of decreased molecular freedom, which is closely related to the suppression of metabolic activity. Therefore, the application of high-ACC SAEW appears to inhibit seed germination, potentially by decelerating the seed’s internal metabolism.

#### 3.2.2 The influence of SAEW on the different phase states of water within rice seeds

**Figure 4:**
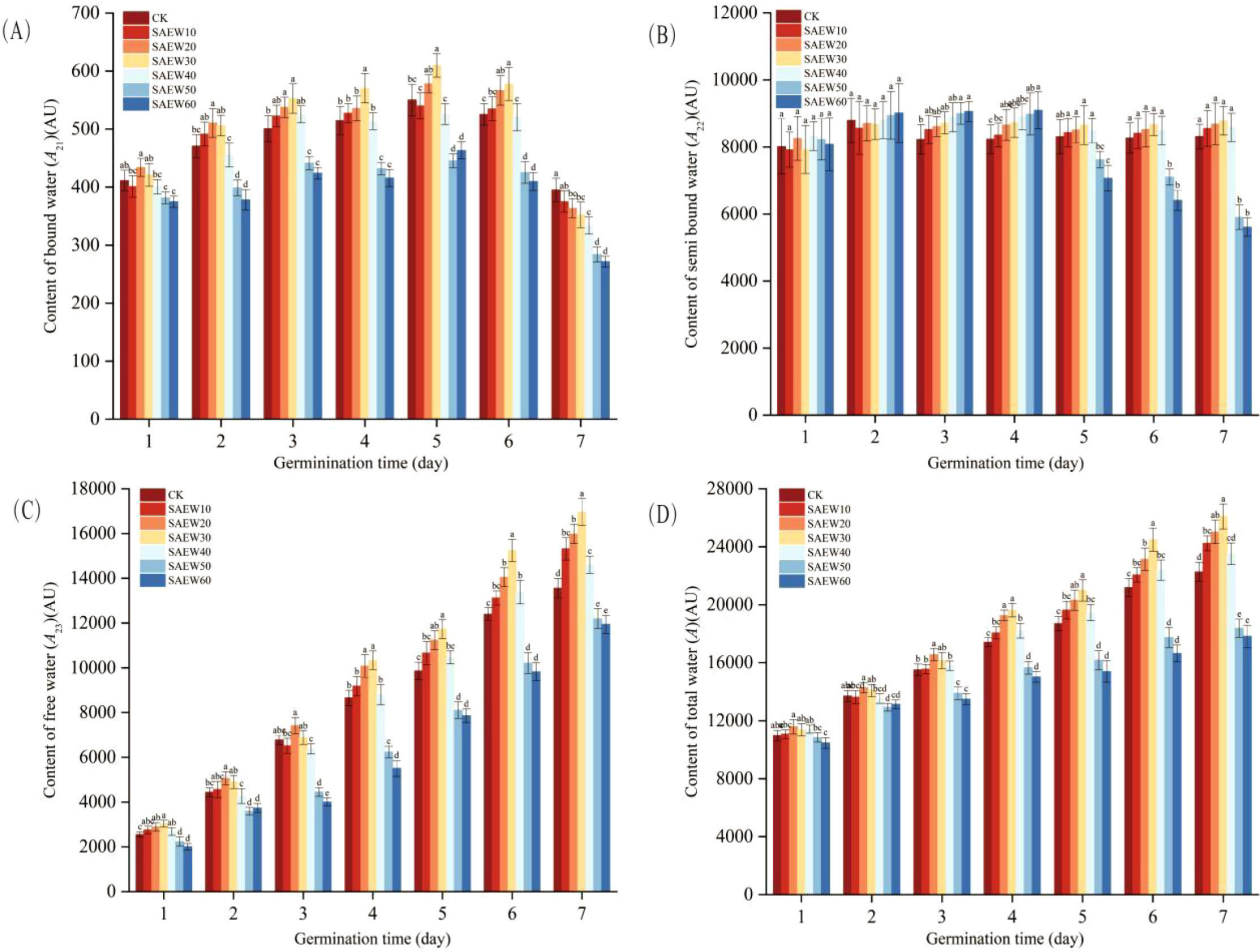
SAEW on water content dynamics in germinating rice seeds (ACC). **(A)** Bound water (*A*_21_), **(B)** semi-bound water (*A*_22_), **(C)** free water (*A*_23_), and **(D)** total water (*A*) peak areas normalized to unit mass. CK: deionized water; SAEW: ACC (10–60 mg/L). Data are mean ± SD. Different letters indicate significant differences between SAEW groups at the same time point (*P* < 0.05).

**Figure 5:**
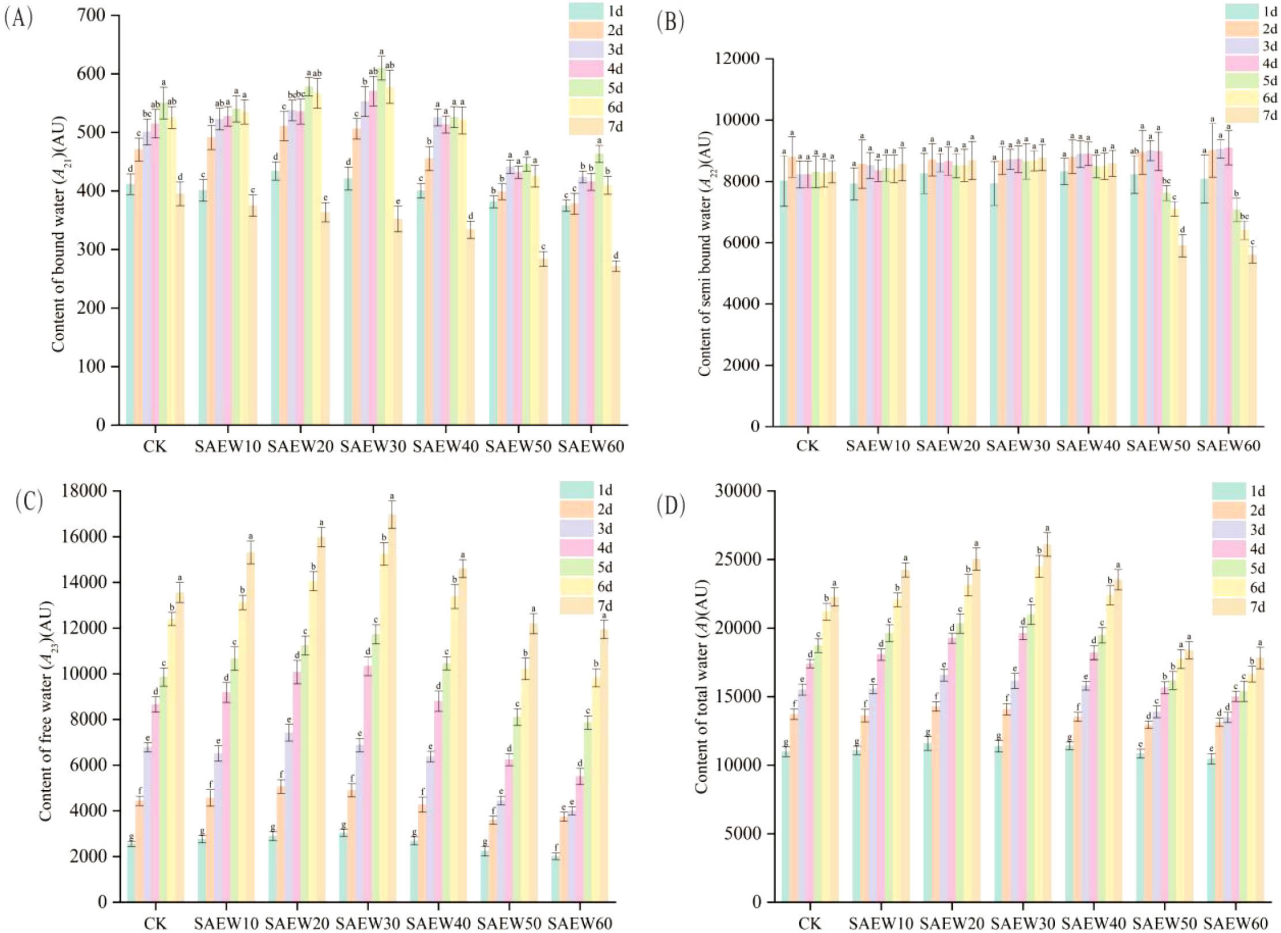
SAEW on water content dynamics in germinating rice seeds (Germination time). **(A)** Bound water (*A*_21_), **(B)** semi-bound water (*A*_22_), **(C)** free water (*A*_23_), and **(D)** total water (*A*) peak areas normalized to unit mass. CK: deionized water; SAEW: ACC (10–60 mg/L). Data are mean ± SD. Different letters indicate significant differences between SAEW groups at the same time point (*P* < 0.05).

The peak areas from each data collection point were statistically analyzed and presented as mean ± standard deviation in the error bar plot shown in **Figure 4** and **Figure 5**. Analysis of **Figure 4** revealed that after treatment with SAEW of different ACC, the content of water in various physical states within the rice seeds continuously changed as germination progressed. Following adequate water uptake, the seeds swelled and germinated, leading to a significant increase in all water populations (*A*_21_, *A*_22_, *A*_23_) and the total water content. SAEW treatments with low and high ACC demonstrated opposing influences on seed water dynamics, manifested by distinct trends in the peak areas of the three water populations. The most pronounced effect of SAEW was observed on the free water (*A*_23_)(*P* < 0.05), followed by a noticeable effect on the bound water (*A*_21_)(*P* < 0.05), and a less marked effect on the semi-bound water (*A*_22_)(*P* < 0.05). Under treatment with low-ACC(10–40 mg/L) SAEW, the peak areas for all water populations showed only minor fluctuations from day 1 to day 7. In contrast, under high-ACC(50–60 mg/L) SAEW treatment, both *A*_21_ and *A*_22_ decreased substantially by day 7, with the mean *A*_21_ value dropping below 300 and the mean *A*_22_ value falling below 4000. Analysis of **Figure 5** indicated that during the later stages of seed germination, the *A*_21_ values of rice seeds treated with low-concentration ACC (10–40 mg/L) SAEW were higher than those of the CK group. The *A*_22_ values of rice seeds treated with high-concentration ACC (50–60 mg/L) SAEW exhibited noticeable fluctuations as germination progressed, while no significant changes were observed in the other groups. Both *A*_23_ and *A* values showed a significant increase over the germination period across all experimental groups(*P* < 0.05).

The variation trend of total water content (*A*) in the six experimental groups and one control group consistently demonstrated the following pattern: the total peak area (*A*) increased sharply during the imbibition stage, grew slowly during the germination stage, and exhibited slight fluctuations in the low ACC groups during the sprouting stage, while the high ACC groups showed only slow growth. Phytohormones can break down stored nutrients such as carbohydrates and proteins in seeds into small molecules, which can be absorbed and utilized by the embryo, thereby breaking seed dormancy and promoting germination (Nguyen et al., 2018). SAEW influences water uptake in rice seeds by affecting the protein bodies. During germination, complex exchanges occur between water components, accompanied by the swelling of protein bodies and changes in storage tissues, leading to the gradual degradation of proteins.

### 3.3 The effect of SAEW treatment on the germination of rice seeds

**Figure 6:**
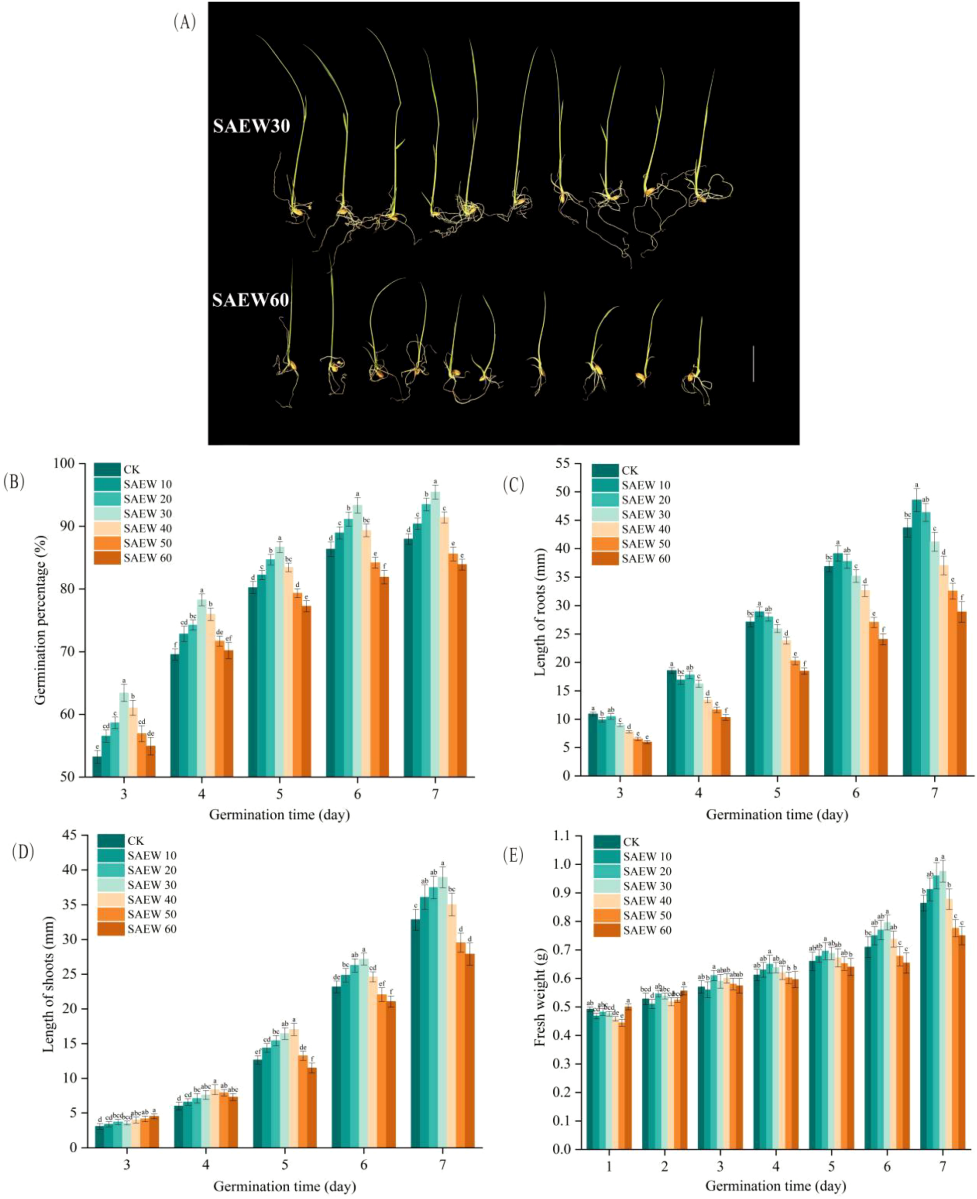
Effects of SAEW on rice seed germination parameters (ACC). **(A)** Comparison between rice seed germination of treatment group SAEW30 and SAEW60. Scale bar, 1 cm. **(B)** Germination percentage, **(C)** Length of roots, **(D)** Length of shoots, and **(E)** Fresh weight. CK: deionized water; SAEW: ACC (10–60 mg/L). Data are mean ± SD. Different letters indicate significant differences between SAEW groups at the same time point (*P* < 0.05).

Germination rate, root length, shoot length, and fresh weight are critical indicators of seed quality and commonly used parameters for evaluating seed vigor. Since seeds transition from the imbibition and activation stages to a stable germination phase on the third day of the germination test—marked by radicle emergence from the seed coat, significant morphological changes, and stable data—measurements of germination rate, root length, and shoot length commence on this day and continue until the test concludes. To fully document the seed imbibition process, fresh weight is measured starting from the first day and continued until the test ends. At the end of the seed germination experiment, a comprehensive comparison of these germination indices was conducted between the best-performing SAEW30 treatment group and the worst-performing SAEW60 treatment group, as shown in **Figure 6A**.

**Figure 7:**
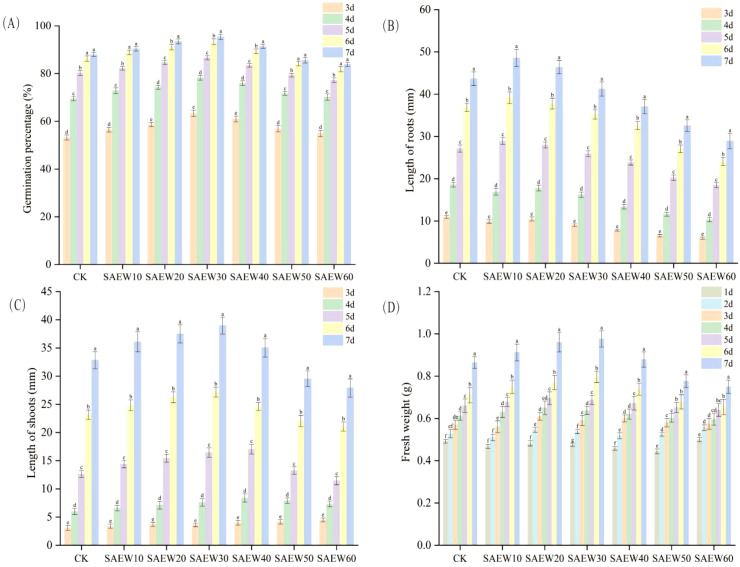
Effects of SAEW on rice seed germination parameters (Germination time). **(A)** Germination percentage, **(B)** Length of roots, **(C)** Length of shoots, and **(D)** Fresh weight. CK: deionized water; SAEW: ACC (10–60 mg/L). Data are mean ± SD. Different letters indicate significant differences between SAEW groups at the same time point (*P* < 0.05).

As shown in **Figure 6B**, SAEW treatment exerted varying degrees of influence on the germination rate of rice seeds. On day 7, the final germination percentages of the SAEW20 and SAEW30 treatment groups both exceeded 90%, which were significantly higher than that of the CK group (by 6.33% and 8.48%, respectively). The SAEW10 and SAEW40 groups also exhibited higher germination percentages than the control (by 2.69% and 3.88%, respectively). In contrast, the SAEW50 and SAEW60 groups showed lower germination percentages compared to the CK group. As shown in **Figure 7A**, the germination percentage of seeds in all groups increased as the germination period progressed. Specifically, for the CK, SAEW10, SAEW50, and SAEW60 treatment groups, no statistically significant change in germination percentage was observed between days 6 and 7(*P ≥* 0.05).

As shown in **Figure 6C**, SAEW treatment exerted varying degrees of influence on root elongation during germination. On day 7, the root lengths of the SAEW10 and SAEW20 treatment groups exceeded that of the CK group (by 11.20% and 6.21%, respectively), whereas the SAEW30, SAEW40, SAEW50, and SAEW60 groups showed shorter root lengths compared to the control. As shown in **Figure 7B**, with the prolongation of the germination period, the root length of seeds in all treatment groups exhibited a significant increase(*P* < 0.05).

As shown in **Figure 6D**, SAEW treatment exerted varying degrees of influence on shoot elongation during germination. Compared to its effect on root length, the response of shoot length differed. On day 7, the shoot lengths of the SAEW10, SAEW20, and SAEW40 groups were all greater than that of the CK group (by 9.86%, 14.17%, and 6.66%, respectively), with the SAEW30 group showing the most pronounced promotion (18.55% greater). In contrast, the SAEW50 and SAEW60 groups exhibited shorter shoot lengths compared to the control. As shown in **Figure 7C**, the shoot length of seeds across all treatment groups increased with the progression of the germination period, with a particularly pronounced growth trend observed between the 5th and 7th days(*P* < 0.05).

As shown in **Figure 6E**, SAEW treatment exerted a significant influence on the fresh weight of rice seeds during germination. On day 7, the SAEW20 and SAEW30 groups exhibited the highest fresh weights, both of which significantly exceeded the CK group.On day 7, the fresh weights of the SAEW10 and SAEW40 groups were comparable to those of the CK group and both were significantly higher than the fresh weights of the SAEW50 and SAEW60 groups. This trend aligns consistently with the results obtained from LF-NMR analysis, indicating a correlation between internal water dynamics and biomass accumulation. As shown in **Figure 7D**, with the extension of germination time, the fresh weight of seeds in each treatment group increased. Notably, a particularly significant growth trend was observed from the 6th to the 7th day of germination(*P* < 0.05).

## 4 Discussion

**Figure 8:**
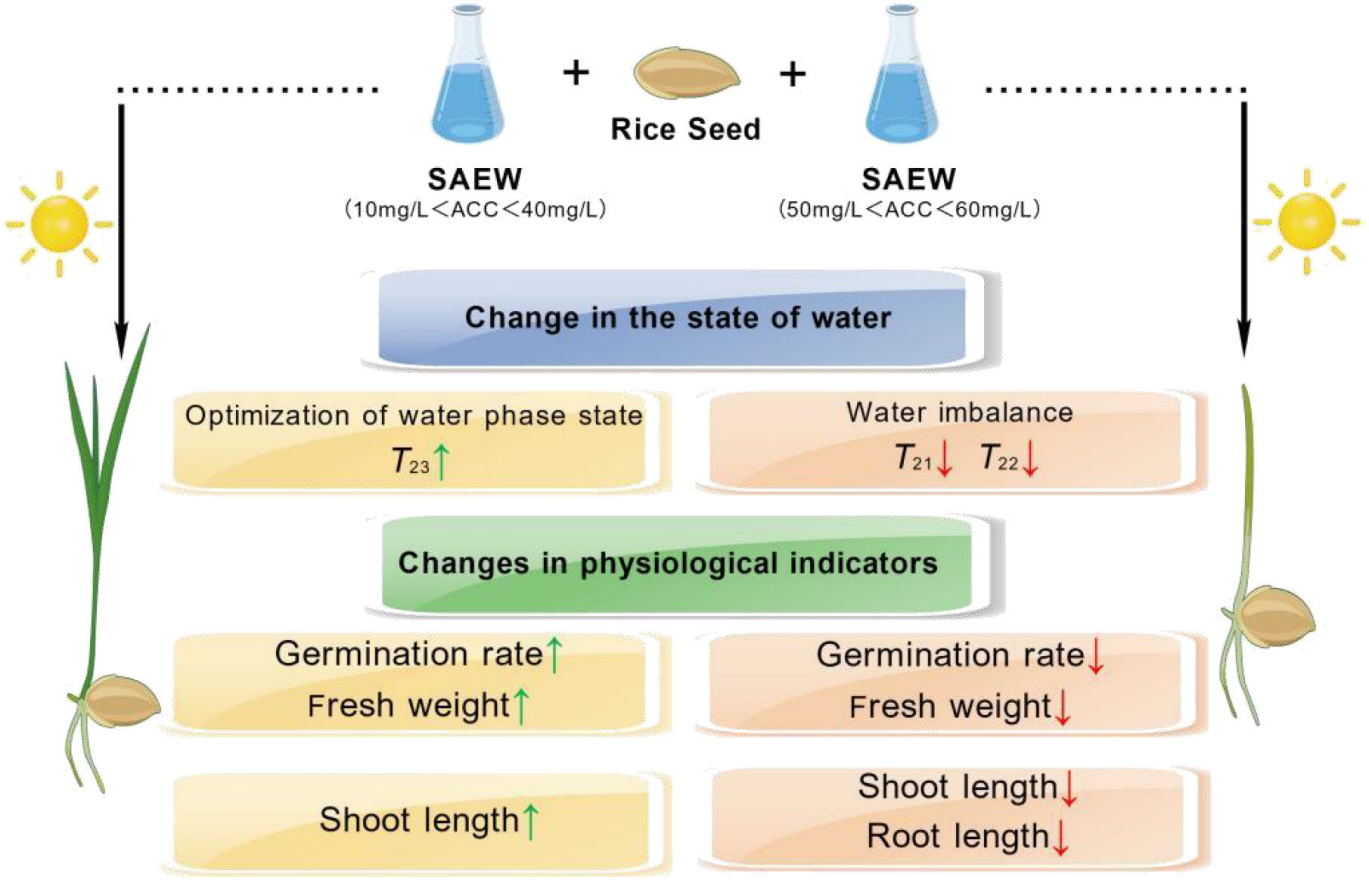
Diagram showing the effect of SAEW on rice seed germination.

In this study, LF-NMR technology combined with a standard germination test was employed to systematically investigate the effects of SAEW with different ACC on water distribution and germination characteristics of rice seeds. The schematic diagram is shown in **Figure 8**. The results not only reveal the dual-effect mechanism of SAEW on seed germination but also elucidate its underlying principles from both biophysiological and biophysical perspectives, providing new insights for a deeper understanding of the application value of this technology in rice breeding.

### 4.1 Concentration-dependent effects of SAEW on seed germination

The results of this study indicate that SAEW has a dual effect on the germination of rice, where low ACC (10–40 mg/L) promotes germination, while high ACC (50–60 mg/L) induces suppression. This biphasic response aligns with electrolyzed water studies reporting redox-mediated physiological shifts in plants (Ding et al., 2015). At the optimal ACC of 30 mg/L, germination rate, shoot length, and fresh weight peaked, coinciding with a significant increase in free water (*T*_23_) observed in LF-NMR spectra.

Low ACC SAEW generates a mild oxidative stress via its suitable ORP, which likely activates redox-sensitive signaling pathways (Liu et al., 2022). This stimulates gibberellin biosynthesis and suppresses abscisic acid activity, breaking seed dormancy (Wu et al., 2022). Concurrently, SAEW enhances membrane permeability, facilitating water uptake and free water accumulation (Ye et al., 2018). The resulting osmotic potential drop drives radicle emergence, as evidenced by MRI pseudo-color images showing extensive high-brightness areas in SAEW30-treated seeds. In contrast, high ACC SAEW induces oxidative damage to cell membranes and cell walls, reducing bound water (*T*_21_) and semi-bound water (*T*_22_) content. This disrupts osmotic balance and inhibits protease activity, corroborating studies where high chlorine concentrations impaired seedling growth (Zhi et al., 2017).

### 4.2 Water kinetics and physiological transitions during germination

LF-NMR and MRI data delineate three germination phases: imbibition (Days 1-2), activation (Days 3-4), and germination (Days 5-7). During imbibition, water infiltrates the seed coat and micropyle, hydrating cytoplasmic colloids—a passive process driven by matrix potential (Li et al., 2023). SAEW accelerates this phase by modifying seed coat hydrophilicity, as seen in the rapid *T*_23_ rise in SAEW30 groups.

The activation phase (Days 3-4) involves protein body swelling and reserve mobilization. SAEW30-treated seeds exhibited rapid protein body expansion, likely due to protease activation by SAEW’s acidic pH (Zhang et al., 2024). This promotes hydrolysis of storage proteins into osmotic regulators and nitrogen sources, reducing cellular osmotic potential and facilitating water influx. Similar phenomena were reported in buckwheat, where SAEW enhanced GABA accumulation via proteolysis (Qiao et al., 2019). The decline in *A*_21_ and *A*_22_ peaks under high ACC indicates membrane integrity loss, aligning with studies on electrolyzed water-induced lipid peroxidation (Liu et al., 2025).

In the germination phase (Days 5-7), free water (*T*_23_) supports metabolic activity and cell elongation. The significant fresh weight increase in SAEW30 groups correlates with *T*_23_ accumulation, reflecting vigorous reserve mobilization. This aligns with LF-NMR studies on Medicago seeds, where free water abundance predicted metabolic vigor (Rao et al., 2022).

### 4.3 Putative physiological mechanisms

SAEW’s germination-promoting effects operate through multi-level mechanisms:

1. Proteolytic Activation: Low ACC SAEW accelerates storage protein degradation via pH and redox-mediated enzyme activation. The resulting amino acids serve as osmotic solutes and nitrogen sources, supporting radicle growth (Liu et al., 2014).
2. Water-State Modulation: SAEW optimizes the bound-to-free water equilibrium. The *T*_23_ shift in SAEW30 seeds indicates enhanced water mobility, critical for metabolite transport and enzyme activity (Li et al., 2022).
3. Hormonal Crosstalk: SAEW may modulate ABA/GA balance, as evidenced by the correlation between free water accumulation and shoot elongation. Reference demonstrated that OsMBF1a regulates GA biosynthesis in rice, a pathway potentially influenced by SAEW’s redox properties (Lou et al., 2025).

### 4.4 Practical implications and limitations

From an agricultural perspective, our findings indicate that treatment with low-ACC SAEW represents a seed pre-sowing technology with multiple advantages: (1) It significantly improves germination uniformity and vigor. (2) Its production cost is low, and it can be rapidly decomposed into harmless components, thereby reducing the risk of environmental pollution (Zhao et al., 2022; Cheng et al., 2023). (3) It is compatible with existing seed treatment protocols.

However, several limitations should be acknowledged: (1) Species-specific responses may exist beyond the cultivars examined in this study. (2) The long-term effects on plant growth and yield remain unexplored. (3) Potential interactions with environmental factors require further investigation.

### 4.5 Future research directions

Based on the aforementioned research findings, we propose that subsequent research priorities should include: (1) Evaluating the genotypic variation in responses to SAEW across diverse rice cultivars; (2) Investigating the hormonal regulatory mechanisms under SAEW treatment, particularly the balance between abscisic acid (ABA) and gibberellins (GA); (3) examining the persistence of SAEW effects throughout the plant life cycle; (4) Exploring potential synergistic interactions with other seed enhancement technologies.

In summary, this study comprehensively demonstrates through experimental evidence that SAEW effectively regulates rice seed germination by modulating water dynamic balance and reserve mobilization. The integration of LF-NMR with conventional germination tests provides a robust methodology for elucidating the biophysical mechanisms underlying seed pretreatment technologies. These findings not only enrich the knowledge system regarding the application of electrolyzed water in agriculture but also offer a scientific basis for optimizing seed treatment strategies.

## 5 Conclusion

1. Low ACC SAEW (10–40 mg/L) promoted rice growth, whereas high ACC SAEW (50–60 mg/L) inhibited it. The germination rates across treatment groups, in descending order, were: SAEW30 > SAEW20 > SAEW10 > SAEW40 > CK > SAEW50 > SAEW60. The trends for shoot length and fresh weight aligned closely with germination outcomes. However, the SAEW10 treatment group exhibited the optimal root length, distinguishing it from other ACC concentrations.
2. Water status changes were analyzed by NMR relaxometry. Based on T_2_ relaxation times, water in rice seeds was classified into three phases: bound water (*T*_21_), semi-bound water (*T*_22_), and free water (*T*_23_). This classification is commonly used to assess seed water dynamics and metabolic activity. Low ACC SAEW (10-40 mg/L) treatments maintained higher levels of all three water phases, indicating better water retention and metabolic support. In contrast, high ACC SAEW (50-60 mg/L) reduced the proportions of bound and semi-bound water while slightly increasing free water, suggesting impaired water binding capacity and potential stress on seed physiology.
3. Pseudo-color processing of proton density-weighted images enabled clear and intuitive observation of water distribution within rice seeds during germination. The germination process of rice seeds was categorized into three distinct stages: imbibition, activation, and sprouting. Following immersion, water entered the seed interior primarily through the seed coat, micropyle, and hilum. Under the influence of SAEW with an appropriate ACC, the decomposition of storage proteins within the seed was accelerated, thereby supplying energy and nutrients to the developing embryo. Among the treatments, SAEW with an ACC of 30 mg/L was identified as the most effective concentration for promoting this process.

## Data Availability

The original contributions presented in the study are included in the article/supplementary material. Further inquiries can be directed to the corresponding author.
